# Patient-specific 3D printed and augmented reality kidney and prostate cancer models: impact on patient education

**DOI:** 10.1186/s41205-019-0041-3

**Published:** 2019-02-19

**Authors:** Nicole Wake, Andrew B. Rosenkrantz, Richard Huang, Katalina U. Park, James S. Wysock, Samir S. Taneja, William C. Huang, Daniel K. Sodickson, Hersh Chandarana

**Affiliations:** 1 Center for Advanced Imaging Innovation and Research (CAI2R) and Bernard and Irene Schwartz Center for Biomedical Imaging, Department of Radiology, NYU Langone Health, NYU School of Medicine, 660 First Avenue, Fourth Floor, New York, NY 10016 USA; 2 Division of Urologic Oncology, Department of Urology, NYU Langone Health, NYU School of Medicine, New York, NY USA

**Keywords:** 3D printing, Augmented reality, Urologic oncology, Kidney cancer, Prostate cancer

## Abstract

**Background:**

Patient-specific 3D models are being used increasingly in medicine for many applications including surgical planning, procedure rehearsal, trainee education, and patient education. To date, experiences on the use of 3D models to facilitate patient understanding of their disease and surgical plan are limited. The purpose of this study was to investigate in the context of renal and prostate cancer the impact of using 3D printed and augmented reality models for patient education.

**Methods:**

Patients with MRI-visible prostate cancer undergoing either robotic assisted radical prostatectomy or focal ablative therapy or patients with renal masses undergoing partial nephrectomy were prospectively enrolled in this IRB approved study (*n* = 200). Patients underwent routine clinical imaging protocols and were randomized to receive pre-operative planning with imaging alone or imaging plus a patient-specific 3D model which was either 3D printed, visualized in AR, or viewed in 3D on a 2D computer monitor. 3D uro-oncologic models were created from the medical imaging data. A 5-point Likert scale survey was administered to patients prior to the surgical procedure to determine understanding of the cancer and treatment plan. If randomized to receive a pre-operative 3D model, the survey was completed twice, before and after viewing the 3D model. In addition, the cohort that received 3D models completed additional questions to compare usefulness of the different forms of visualization of the 3D models. Survey responses for each of the 3D model groups were compared using the Mann-Whitney and Wilcoxan rank-sum tests.

**Results:**

All 200 patients completed the survey after reviewing their cases with their surgeons using imaging only. 127 patients completed the 5-point Likert scale survey regarding understanding of disease and surgical procedure twice, once with imaging and again after reviewing imaging plus a 3D model. Patients had a greater understanding using 3D printed models versus imaging for all measures including comprehension of disease, cancer size, cancer location, treatment plan, and the comfort level regarding the treatment plan (range 4.60–4.78/5 vs. 4.06–4.49/5, *p* < 0.05).

**Conclusions:**

All types of patient-specific 3D models were reported to be valuable for patient education. Out of the three advanced imaging methods, the 3D printed models helped patients to have the greatest understanding of their anatomy, disease, tumor characteristics, and surgical procedure.

## Background

Navigating a cancer diagnosis and making decisions about cancer treatment can be challenging for many patients. Individual treatment plans vary and depend on the type of cancer, stage of the disease, and other comorbidities. Recently, there has been a clear move towards shared decision making and patients want to assume an increasing role in medical decision making, with 92.5% of men with newly diagnosed prostate cancer wanting to play either an active or a collaborative role in decision making with their physician [1].

For patients undergoing major urological procedures, pre-operative imaging plays a critical role in patient counseling and shared surgical decision making [2–5]. At our institution, urologic surgeons often use 2D images during patient consultation, however we speculate that many patients have a difficult time conceptualizing these images. In order to make decisions regarding treatment options, it is imperative that patients are provided with an adequate amount of information to understand their disease and treatment plan.

To date, experiences on the use of 3D models to facilitate patient understanding in the context of urologic oncology are limited to the small 3D printing case studies described below [6–8]. For renal cancer, Silberstein et al. anecdotally reported that for a set of five 3D printed renal cancer models, patients and their families felt that 3D models enhanced their comprehension of the tumor anatomy in relation to the surrounding structures and helped to improve the goals of the surgery [6]. Next, in a pilot study of seven patients, Bernard et al. created personalized 3D printed kidney tumor models as a useful tool for patient education and demonstrated an improvement in understanding of basic kidney physiology (16.5%), kidney anatomy (50%), tumor characteristics (39.3%), and the planned surgical procedure (44.6%) [7]. Porpiglia et al. created 3D printed models for 8 patients undergoing robotic-assisted radical prostatectomy and 10 undergoing robotic-assisted radical partial nephrectomy and reported that patients responded favorably about the use of the technology during case discussion with the surgeon [8]. Finally, Schmit et al. evaluated the use of 3D printed models on patients understanding of renal cryoablation; and although they found no improvement of patients’ objective anatomy and procedural knowledge with 3D models, patients’ perceived value of the 3D models [9].

While these small studies above support the added benefit of 3D models, the role that 3D models can play in shared decision making is yet to be defined. We believe that in addition to 3D printed models, advanced visualization of medical images in 3D formats such as virtual reality (VR), augmented reality (AR), or 3D computer models might also help to overcome the limitations of consultations performed with 2D images. All types of 3D models could be referred to during the consultation and could be used to describe the anatomy, disease, and treatment options allowing for improved levels of patient understanding of anatomy and disease, as well as facilitate better patient decisions regarding the treatment plan. The aim of this study was two-fold: (1) to prospectively evaluate, in a large cohort of patients, the usefulness of patient-specific 3D urologic oncology (kidney and prostate cancer) models for patient education and (2) to compare the usefulness of different types of 3D models in patient education.

## Materials and methods

Patients with magnetic resonance imaging (MRI)-visible prostate cancer (PI-RADS v2 score ≥ 3) and biopsy confirmed cancer undergoing either robotic assisted radical prostatectomy or focal ablative therapy or patients with renal masses (nephrometry score (NS) ≥ 7, diameter ≥ 4 cm, or polar lesions) undergoing partial nephrectomy were prospectively enrolled in this IRB approved study (*n* = 200). Of the 200 total patients, 151 had prostate cancer: 104 patients with 146 lesions underwent prostatectomy and 47 patients with 69 lesions underwent focal ablative therapy. The breakdown of PI-RADS scores was as follows: PI-RADS 2 = 28, PI-RADS 3 = 68, PI-RADS 4 = 82, PI-RADS 5 = 28, and no PI-RADS could be assigned in 9 cases with biopsy confirmed prostate cancer in the region of the MR defined lesion. There were 49 patients with kidney cancer (29 males and 20 females) with the following NS breakdown: NS 4 = 2, NS 5 = 2, NS 6 = 7, NS 7 = 14, NS 8 = 13, NS 9 = 8, NS 10 = 3. The mean age and range was 63.64 ± 8.22 years. Patients underwent routine clinical imaging protocols and were randomized to receive pre-operative planning with imaging alone or imaging plus a patient-specific 3D model which was either 3D printed, visualized in AR, or viewed in 3D on a 2D computer monitor.

### Image acquisition

Images for all patients were acquired according to the clinical protocol. For prostate cancer patients, multi-parametric MRI was performed on a 3 T MRI system. A 3D turbo spin-echo T2-weighted imaging sequence (i.e. SPACE) with a spatial resolution of 0.6 × 0.6 × 1 mm, a diffusion-weighted imaging (DWI) sequence, and a dynamic contrast-enhanced sequence were utilized for generation of the 3D model. For kidney cancer patients, images were acquired on a 1.5 T MR system (Avanto, Siemens, Erlangen, Germany) using a phased array body coil or multi-detector row computed tomography (CT) system (Somatom Definition Edge or Force, Siemens, Erlangen, Germany). T1-weighted fat-saturated gradient echo (GRE) images in different phases of contrast enhancement were used for model generation. The standard representative MR sequence parameters are: TR = 3.58 ms, TE = 1.3 ms, FA = 12°, an interpolated spatial resolution of 1.4 mm × 1.4 mm × 2 mm, and breath-hold acquisition time ranged from 13 to 20 s. The standard dual phase CT protocol included pre- and post-contrast imaging in the nephrographic phase. Axial images were acquired with a 0.625 mm slice thickness (120kVp, 150mAs, 512 × 512 matrix) and sagittal and coronal images were reconstructed with a slice thickness of 3–4 mm.

### 3D modeling

**Fig. 1:**
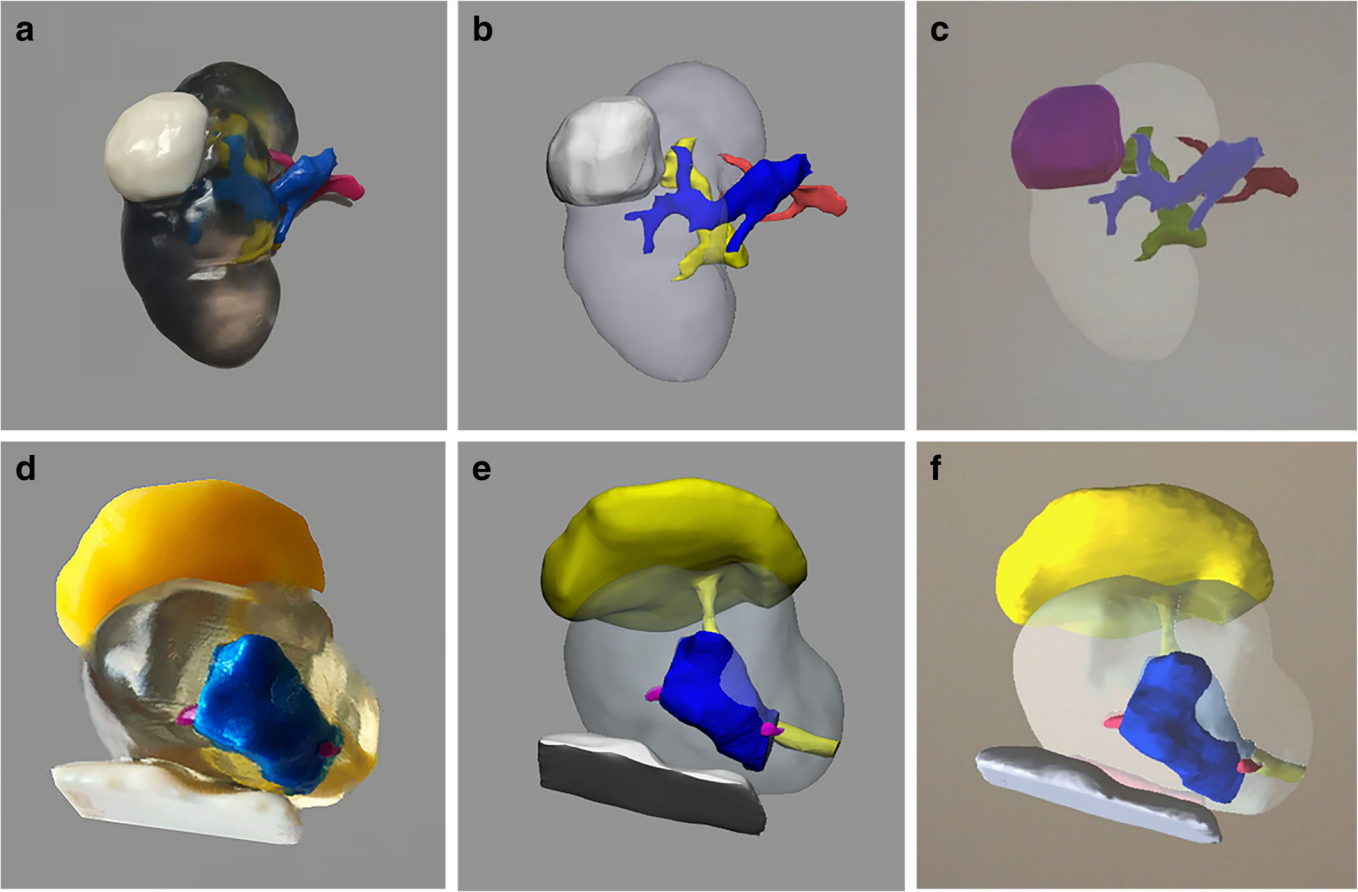
(**a**) 3D printed, (**b**) 3D computer, and (**c**) AR kidney cancer models with the kidney – clear, tumor –white (3D print and computer), tumor – purple (AR), artery – red, vein – blue, collecting system – yellow. (**d**) 3D printed, (**e**) 3D computer, and (**f**) AR prostate cancer models (sagittal view) with the prostate –clear, tumor – blue, rectal wall – white, bladder neck and urethra – yellow, and neurovascular bundles –pink

Image segmentation of the urologic cancer models was performed using Mimics 20.0 (Materialise, Leuven, Belgium) as described previously [10]. For kidney cancer models, the kidney, tumor, vein, artery, and collecting system were segmented and for prostate cancer models, the prostate, tumor, rectal wall, urethra and bladder neck, and neurovascular bundles were segmented. Each segmented region of interest raster was converted to a surface mesh which could be exported in 3D PDF format for direct visualization, converted to standard tessellation language (.stl) format for multi-colored 3D printing (J750, Stratasys, Eden Prairie, MN), or converted to Alias/Wavefront (.obj) format for AR programming and visualization using the Microsoft HoloLens AR device [11]. Figure 1 shows representative 3D models of each type.

### 3D model analysis

A 5-point Likert scale survey was administered to patients prior to the surgical procedure to determine understanding of the cancer and treatment plan as described in Table 1. If randomized to receive a pre-operative 3D model, the survey was completed twice, before and after viewing the 3D model.

**Table 1:** Likert-scale survey to assess patient understanding of disease and procedure

Question	Answer
1. How would you rate your understanding of your cancer/disease?	1 – Very poor
	2 – Poor
	3 – Fair
	4 – Good
	5 – Very good
2. I understand how big my cancer/tumor is.	1 – Strongly disagree
	2 – Disagree
	3 – Neutral
	4 – Agree
	5 – Strongly agree
3. I understand where my cancer/tumor is located.	1 – Strongly disagree
	2 – Disagree
	3 – Neutral
	4 – Agree
	5 – Strongly agree
4. I understand why my surgeon chose the treatment plan being offered.	1 – Strongly disagree
	2 – Disagree
	3 – Neutral
	4 – Agree
	5 – Strongly agree
5. I feel comfortable with the surgical plan.	1 – Strongly disagree
	2 – Disagree
	3 – Neutral
	4 – Agree
	5 – Strongly agree

Survey responses for each of the 3D model groups were compared to the group with just imaging using the Mann-Whitney test. The paired-sample Wilcoxan signed rank test was utilized to compare results for patients who answered the surveys twice, before and after seeing a 3D model. In addition, the cohort that received 3D models completed additional questions to compare usefulness of the different forms of visualization of the 3D models (Table 2). Results for the 3D printed models were compared to AR and 3D computer models using the Mann-Whitney test. Statistical analyses were performed in SPSS Statistics Version 23 (IBM Corp, Armonk, NY) and Matlab R2017a (The Mathworks Inc., Natick, MA).

**Table 2:**
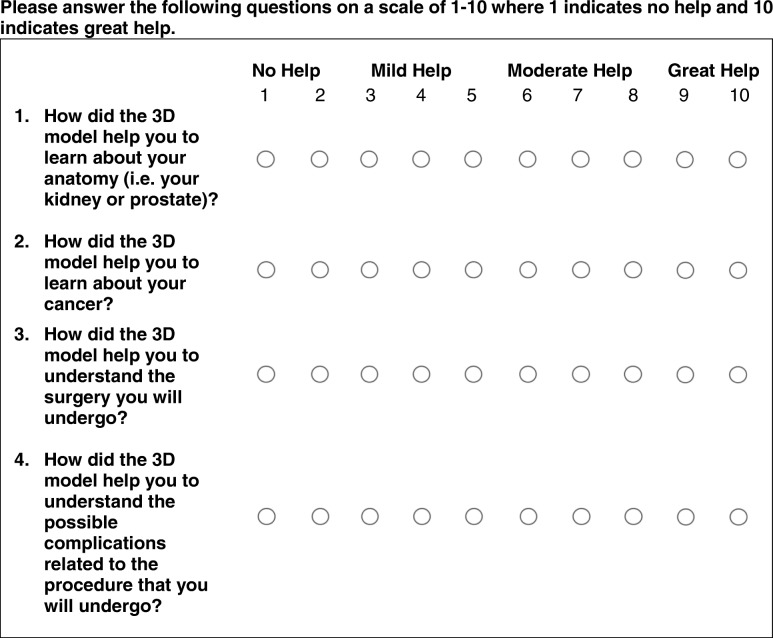
Survey to assess patient perceived usefulness of 3D models

## Results

All 200 patients completed the survey after reviewing their cases with their surgeons using imaging only. 127 patients completed the 5-point Likert scale survey regarding understanding of disease and surgical procedure twice, once with imaging and again after reviewing imaging plus a 3D model. Overall, the 3D printed models performed better than imaging, 3D computer models, and AR models (Table 3). Patients had a greater understanding using 3D printed models versus imaging for all measures including comprehension of disease (4.70 ± 0.54, *p* < 0.001), cancer size (4.60 ± 0.54, *p* < 0.001), cancer location (4.75 ± 0.50, *p* < 0.001), treatment plan (4.78 ± 0.45, *p* < 0.001), and comfort level regarding the treatment plan (4.69 ± 0.57, *p* = 0.013). Patients also had a greater understanding of their anatomy and disease as well as improved comfort level using 3D printed models as compared to AR models (range 4.60–4.70/5 vs 3.50–4.23/5, *p* < 0.05). There was no improvement in understanding for any of the measures for the AR model group as compared to the imaging group or the 3D printed versus computer model groups.

**Table 3:** Likert scale survey responses for understanding of cancer/disease, tumor size, tumor location, treatment plan, and comfort level. Bold values with a * next to the value indicates statistically significant improvement with the 3D model (*p* < 0.05)

	Imaging (*n* = 200)	3D Printed Model (*n* = 55)	AR Model (*n* = 26)	3D Computer Model (*n* = 46)
Disease	4.28 ± 0.80	4.70 ± 0.54*	4.23 ± 0.59	4.50 ± 0.66
Cancer Size	4.06 ± 0.91	4.60 ± 0.54*	4.04 ± 0.92	4.48 ± 0.59*
Cancer Location	4.34 ± 0.69	4.75 ± 0.50*	4.23 ± 0.82	4.65 ± 0.48*
Treatment Plan	4.49 ± 0.62	4.78 ± 0.45*	4.35 ± 0.85	4.70 ± 0.47*
Comfort Level	4.40 ± 0.76	4.69 ± 0.57*	3.50 ± 1.97*	4.53 ± 0.61

Stratified by cancer type, both prostate cancer and kidney cancer patients had the highest level of understanding with the 3D printed models (Table 4). For the prostate cancer patients, there was statistical significance with 3D printed models as compared to imaging for understanding of disease (*p* < 0.001), cancer size (*p* < 0.001), cancer location (*p* < 0.001), and treatment plan (*p* = 0.007). Patient understanding was greater regarding cancer size (*p* = 0.018) and location (*p* = 0.011) with 3D computer models versus imaging. For the kidney cancer patients, statistical significance was seen with 3D printed models as compared to imaging for questions regarding cancer size (*p* = 0.04), cancer location (*p* = 0.012), treatment plan (*p* = 0.014), and comfort level (*p* = 0.028). There was no difference in level of understanding with the AR models as compared to imaging or with the 3D computer models versus imaging for the kidney cancer cohort.

**Table 4:** Likert scale survey responses stratified by cancer type

	Imaging	3D Printed Model	AR Model	3D Computer Model
Disease				
Prostate Cancer	4.34 ± 0.77	4.81 ± 0.40	4.22 ± 0.60	4.50 ± 0.58
Kidney Cancer	4.12 ± 0.84	4.53 ± 0.72	N/A	4.53 ± 0.80
Cancer Size				
Prostate Cancer	3.98 ± 0.94	4.57 ± 0.55	4.00 ± 0.95	4.43 ± 0.63
Kidney Cancer	4.30 ± 0.74	4.71 ± 0.47	N/A	4.59 ± 0.51
Cancer Location				
Prostate Cancer	4.28 ± 0.72	4.70 ± 0.51	4.22 ± 0.85	4.64 ± 0.49
Kidney Cancer	4.52 ± 0.54	4.88 ± 0.33	N/A	4.71 ± 0.47
Treatment Plan				
Prostate Cancer	4.47 ± 0.64	4.76 ± 0.49	4.35 ± 0.88	4.71 ± 0.46
Kidney Cancer	4.53 ± 0.54	4.88 ± 0.33	N/A	4.71 ± 0.47
Comfort Level				
Prostate Cancer	4.43 ± 0.70	4.60 ± 0.65	3.50 ± 1.97	4.60 ± 0.58
Kidney Cancer	4.28 ± 0.86	4.86 ± 0.36	N/A	4.36 ± 0.67

**Fig. 2:**
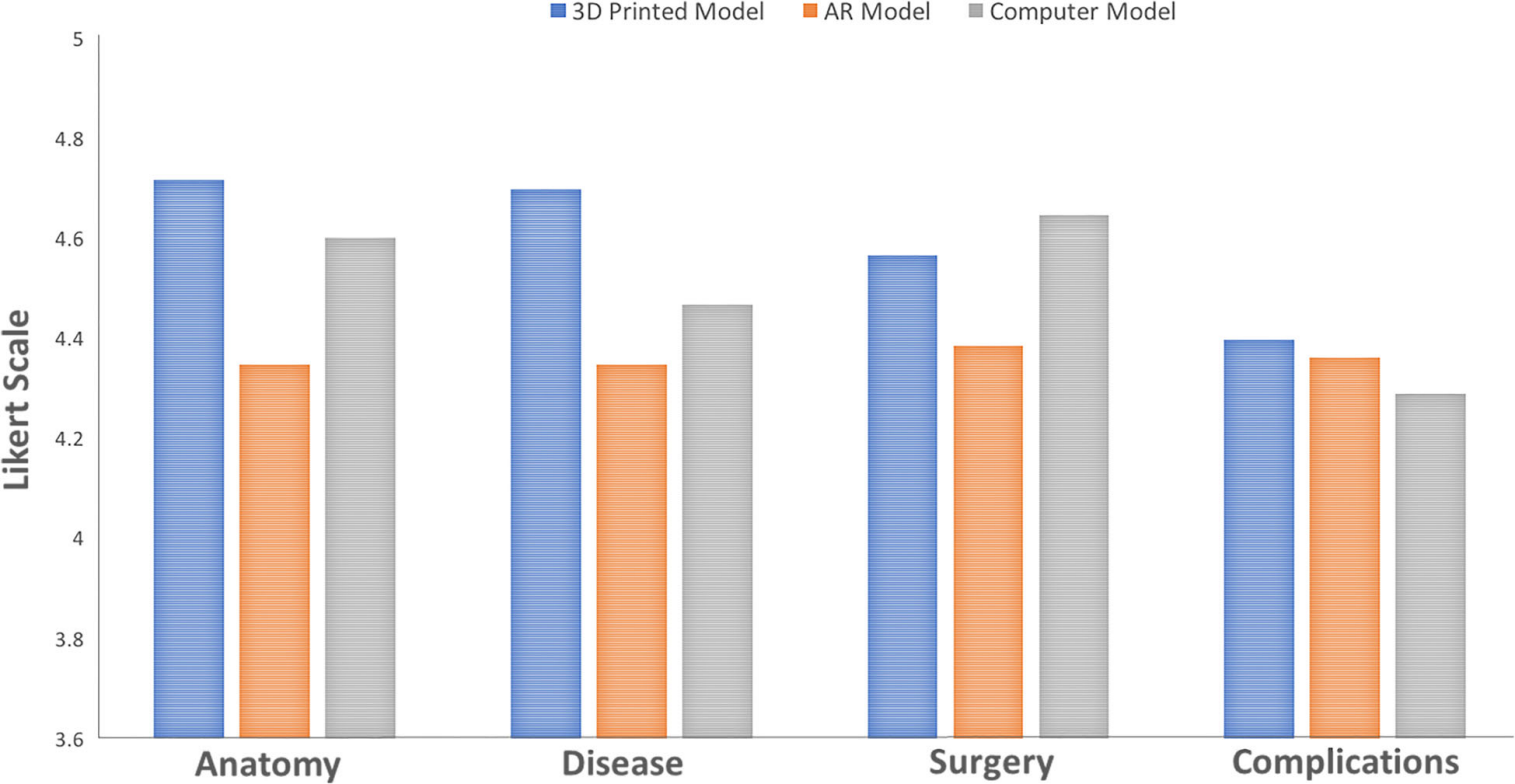
Aggregated survey responses comparing usefulness of different 3D visualization methods in understanding various metrics. Circled values indicate statistical significance between groups (*p* < 0.05)

Results for the second survey questions assessing patient perceived usefulness of 3D models are shown in Fig. 2. 89 patients completed this additional survey: 38 with 3D printed models, 12 with AR models, and 39 with 3D computer models. All models were reported to be useful on the 10-point scale with results for 3D printed models ranging from 8.45–9.21/10, AR models from 7.50–7.92/10, and 3D computer models from 7.95–8.92/10. Similar to the findings above, the 3D printed models performed the best for all questions. Patients found the 3D printed models to be more helpful than the AR models with respect to their comprehension of anatomy (9.21 ± 1.49 vs 7.92 ± 2.84, *p* = 0.04). In addition, patients noted the 3D printed models to be more valuable than both AR and 3D computer models in regards to their disease understanding (9.11 ± 1.86 vs 7.50 ± 3.35 vs 8.59 ± 2.05, *p* < 0.05). AR and 3D computer models were reported to be equally helpful with respect to all questions.

For this cohort of patients with renal cancer, the 3D printed model helped one patient decide to undergo robotic-assisted partial nephrectomy instead of forgoing the procedure and the 3D model helped a second patient to decide between radical and partial nephrectomy, the preferred procedure since part of the organ could be spared. In general, for prostate cancer patients, if the cancerous lesion was located in close proximity to the neurovascular bundles, then the patient-specific 3D model helped the patient to better understand why the nerve could not be preserved.

## Discussion/conclusions

At our institution, consultations for patients with kidney and prostate malignancies are routinely performed using imaging only to explain the disease and surgical procedure. We have previously demonstrated that patient-specific 3D printed models of renal malignancies influence pre-surgical planning decisions [12]. In addition, 3D printed models can facilitate nerve-sparing prostatectomy [13].

Outside the field of urologic malignancies, Biglino et al. showed that 3D models are useful tools for engaging parents of children with congenital heart disease (*n* = 103) in discussions with their surgeon [14]. Also, in a small cohort of 20 patients 15–18 years olds with congenital heart disease, Biglino et al. demonstrated that 3D models helped their understanding of anatomy and improved their visit [15]. Another small study by Yang et al. reported that 3D printed liver models improved parental understanding of basic liver anatomy and physiology, tumor characteristics, the planned surgical procedure, and surgical risks for seven children with hepatic tumors scheduled for hepatectomy [16]. Van de Belt et al. showed that 3D printed models aid in education for a small cohort of 11 patients with glioma. Patients reported that it was easier to ask their surgeon questions based on their personalized model and that it supported their decision about preferred treatment [17]. Finally, Sander et al. created a single 3D printed educational model of the nasal sinus and performed a randomized, prospective study where 50 surgical candidates were given the explanation of their anatomy, disease state, and treatment options with the model and 50 without a model as a control group; and they found statistically significant improvements in understanding of treatment options, anatomy, and disease with the 3D model [18].

In this study, we evaluated how 3D models of renal and prostate cancer can impact patient education. Patients reported that all types of the 3D models were helpful in learning about the anatomy, disease, cancer location, and treatment plan. Overall, 3D printed models were reported to be the most helpful and showed the greatest improvement in patient understanding. 3D computer models also improved patient understanding of their cancer and surgical procedure compared to imaging only. Although AR models were reported to be valuable by the patients, they did not increase patient understanding in regards to the anatomy, disease, or treatment choice.

Our findings that 3D printed models of renal and prostate malignancies are useful tools for patient education and surgical decision making are consistent with findings by Silberstein, Bernhard, and Porpiglia [6–8]. However, there are number of major differences in our study compared to these previous studies. The first is that our study included a much larger cohort of patients. Next, our study included patients with prostate cancer which have not been studied before. Finally, we tested how 3D printed models performed compared to other methods of 3D modeling including AR models and 3D computer models. To our knowledge this is the largest study evaluating the use of 3D models for patient education and the first study to report on how different types of 3D models may influence patient education.

As compared to traditional imaging or other methods of advanced imaging visualization such as 3D computer models or AR, we believe that 3D printed anatomical models allow for enhanced insight into the underlying anatomy since they provide both spatial comprehension and tactile feedback. Specifically, combining multisensory inputs of touch and vision leads to improved spatial conceptualization versus simply visualizing one’s own anatomy in 3D as a computer model or AR model. In addition, to-scale 3D printed anatomical models allow for one to comprehend the true size of an organ, the cancer, as well as other pertinent anatomical structures. This comprehension of size and scale is difficult to replicate in 3D computer or AR models that can be zoomed in and out to be any size.

One limitation of this study is that the patient questionnaires with imaging was performed first followed by the 3D models. Receiving the information a second time with the addition of a 3D model may improve understanding due to repetition rather than due to the use of 3D models. However, the comparison between various types of 3D model is still valid as patients were randomized to receive 3D printed models, AR models, or 3D computer models. 3D printed models are obviously more costly compared to AR or computer models. This study did not include a detailed cost-analysis, as it focused on assessing the usefulness of personalized 3D models on patient understanding.

In conclusion, although all types of patient-specific 3D models were reported to be useful for patient education, the 3D printed models had the largest improvement in patient understanding of anatomy, disease, tumor characteristics, and the surgical procedure.

## Abbreviations

2D: Two-dimensional
3D: Three-dimensional
AR: Augmented Reality
MRI: Magnetic Resonance Imaging
obj: Alias/Wavefront Format
stl: Standard Tesselation Language/Standard Triangle Language
VR: Virtual Reality
